# Erucic acid improves the progress of pregnancy complicated with systemic lupus erythematosus by inhibiting the effector function of CD8^+^ T cells

**DOI:** 10.1002/mco2.382

**Published:** 2023-09-26

**Authors:** Yanling Chang, Meng Jiang, You Wang, Qiong Fu, Sihan Lin, Jiayue Wu, Wen Di

**Affiliations:** ^1^ Department of Obstetrics and Gynecology Renji Hospital School of Medicine Shanghai Jiao Tong University Shanghai China; ^2^ Department of Obstetrics and Gynecology Shanghai Key Laboratory of Gynecologic Oncology Shanghai China; ^3^ Department of Rheumatology Renji Hospital School of Medicine Shanghai Jiao Tong University Shanghai China; ^4^ Shanghai Institute of Rheumatology Shanghai China; ^5^ Department of Obstetrics and Gynecology, State Key Laboratory of Oncogenes and Related Genes Shanghai Cancer Institute Renji Hospital School of Medicine Shanghai Jiao Tong University Shanghai China

**Keywords:** CD8^+^ T cells, erucic acid, pregnancy, systemic lupus erythematosus

## Abstract

Pathogenic CD8^+^ T cells are pivotal contributors to the onset of systemic lupus erythematosus (SLE). Erucic acid (EA) has been proven to have anti‐inflammatory activity. However, the capacity of EA to regulate pathogenic CD8^+^ T cells in the context of pregnancy complicated with SLE (pSLE) remains unclear. In our investigation, we observed augmented CD8^+^ T cell effector function juxtaposed with diminished EA levels in pSLE patients relative to healthy pregnant controls. Significantly, plasma EA levels exhibited a negative correlation with the severity of pSLE‐associated complications. In blood from patients with pSLE, EA inhibited the effector function of CD8^+^ T cells, concurrently dampening the maintenance of stem cell‐like memory CD8^+^ T cells. Mechanistically, EA orchestrated the inhibition of CD8^+^ T cell effector function by impeding signal transducer and activator of transcription 3 phosphorylation and promoting ferroptosis. Moreover, EA supplementation in pregnant MRL/lpr mice manifested as the attenuation of uterine CD8^+^ T cell effector function, culminating in the mitigation of placental pathological damage. Our findings uncover the immune response modulatory effects of EA upon pathogenic CD8^+^ cells, thereby unveiling new perspectives for therapeutic strategies targeting pSLE patients.

## Graphical abstract

- The effector function of CD8+ T cells in peripheral blood and decidua of patients with pregnancy complicated with systemic lupus erythematosus (pSLE) was significantly enhanced.
- Erucic acid (EA) levels in plasma and decidua were decreased in patients with pSLE.
- EA can inhibit CD8+ Tcells effector function by targeting the Signal transducer and activator of transcription 3 (STAT3)‐ferroptosis negative feedback in patients with pSLE.
- EA supplementation alleviates placental pathological damage in lupus mice.

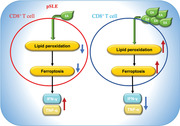

## 1 INTRODUCTION

Systemic lupus erythematosus (SLE) is an autoimmune disease that affects multiple organs and produces autoantibodies. It is more prevalent in females and commonly occurs in women of childbearing age.^1^, ^2^ Women with SLE who become pregnant face a substantially greater risk of difficult pregnancies and adverse pregnancy outcomes, including fetal loss, preterm birth, fetal growth restriction, and preeclampsia.^3^ Despite marked advances in diagnostic and therapeutic strategies to manage SLE, the incidence of placental pathology during pregnancy complicated with SLE (pSLE) is more elevated, compared to the public at large, and around 20%−25% of patients suffering from pSLE will have adverse pregnancy outcomes.^4^ The pathological mechanisms that complicate pSLE have not been thoroughly studied; there are still no effective approaches to predict or to treat complications during pSLE. Therefore, the identification of effective methods to predict complications associated with pSLE as well as therapeutic modalities to alleviate observed complications during pSLE represent unmet medical needs.

CD8^+^ T cells are critical for the development of SLE, affecting pSLE mainly through increased infiltration in tissues, phenotypic changes, and enhanced effector functions that can induce pathological pregnancy outcomes.^5^, ^6^, ^7^, ^8^, ^9^ CD8^+^ T cells are able to produce inflammatory cytokines such as interferon‐gamma (IFN‐γ) and tumor necrosis factor‐alpha(TNF‐α), and the regulation of their effector functions by proinflammatory events is a key factor contributing to pregnancy complications.^10^ CD8^+^ T cells can differentiate into the stem cell‐like memory (Tscm), which is characterized bylong‐lived, self‐renewal capacity, and constant production of cytokines, and they serve as precursors of effector CD8^+^ T cells.^11^, ^12^

Fatty acid (FA) metabolism regulates T‐cell activation and survival and thus has an important role in autoimmune disease.^13^, ^14^ Erucic acid (EA), is a long‐chain FA with one unsaturated position at the omega‐9 position. EA can be consumed in cruciferous vegetables, mustard, canola oil, and abyssal fish,^15^ making it easy to incorporate in most diets. EA acts by preventing the overactivation of inflammatory signals, reducing the enlistment of cytotoxic CD8^+^ T cells throughout virus‐infected lungs.^16^ Signal transducer and activator of transcription 3 (STAT3) acts as a transcription factor, promoting T‐cell persistence. Its activation governs CD8^+^ T‐cell lipid peroxidation as well as ferroptosis.^17^, ^18^ CD36 is a cell surface glycoprotein that binds to FAs and facilitates FA transport for lipid utilization.^19^ The expression of CD36 on CD8^+^ T cells impacts its effector function. Elevated expression of CD36 increases cell death of CD8^+^ T cells during anti‐tumor responses.^20^ The FA reliance of CD8^+^ T cells allows them to lower the production of inflammatory cytokines, providing a novel target for pSLE treatment.

In this study, we found reduced EA levels and increased CD8^+^ T‐cell effector function in pSLE patients. EA abolished the effector function of CD8^+^ T cells through the reduction of STAT3 phosphorylation and promoting ferroptosis. In a mouse model of SLE disease, pregnant mice that received EA supplementation presented with diminished effector performance of uterine CD8^+^ T cells and reduced pathological placental injury. These results indicate the pathogenic function of CD8^+^ T cells in pSLE patients and that EA can regulate CD8^+^ T cells, thus indicating a potential therapeutic strategy for patients with pSLE.

## 2 RESULTS

### 2.1 CD8^+^ Tcell effector function is increased in patients with pSLE

**FIGURE 1:**
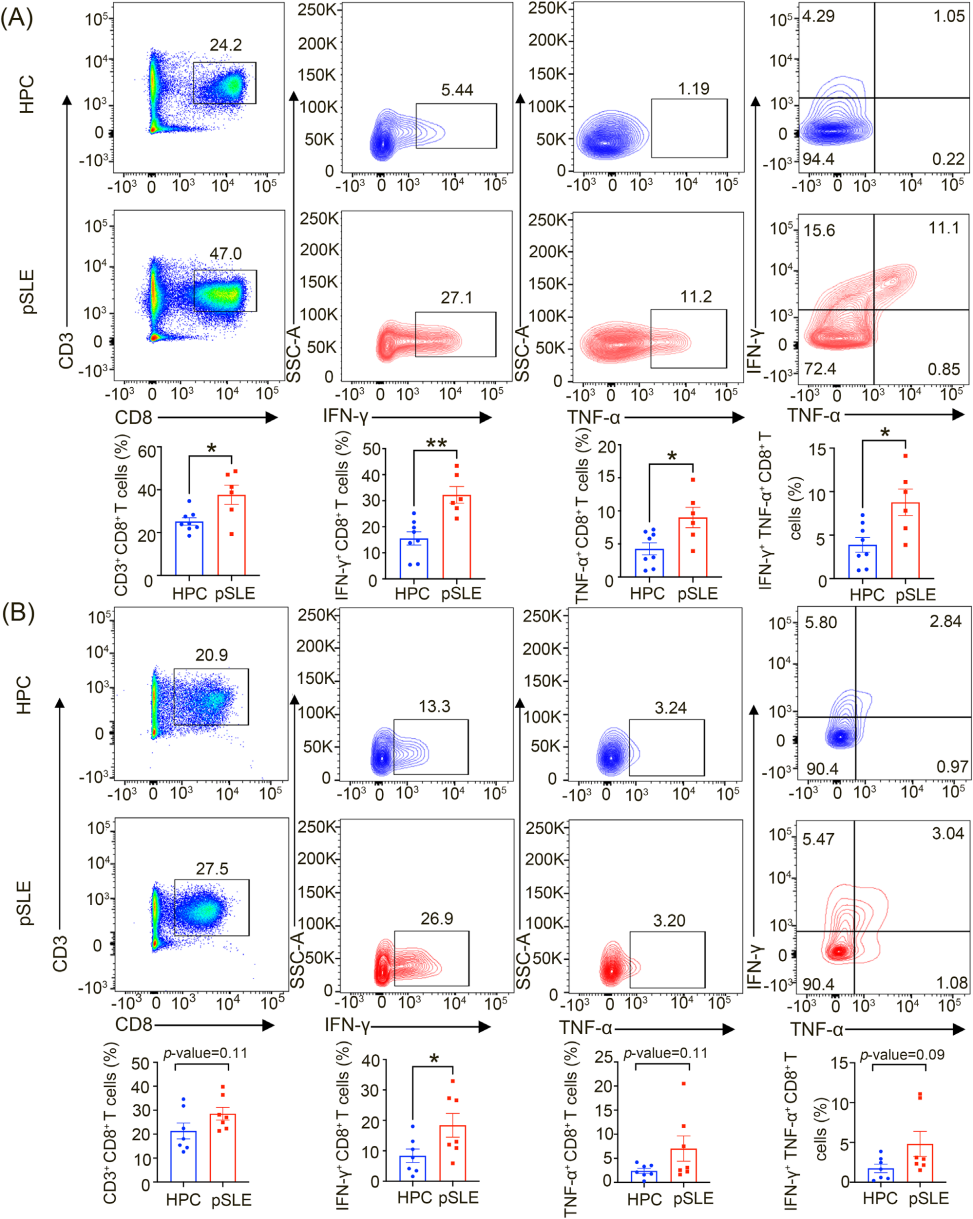
Proportion and effect function of CD8^+^ T cells within peripheral blood and decidua obtained from patients with pregnancy complicated with systemic lupus erythematosus (pSLE) and healthy pregnant control (HPC). (A) Proportion of CD3^+^ CD8^+^ T cells and production of interferon‐gamma (IFN‐γ), tumor necrosis factor‐alpha (TNF‐α), IFN‐γ^+^ TNF‐α^+^ CD8^+^ T cells throughout peripheral blood from patients with pSLE (*n* = 6) and HPC (*n* = 8) were contrasted. (B) Proportion and effector function of CD3^+^ CD8^+^ T cells were compared across decidua from pSLE patients (*n* = 7) and HPC (*n* = 7). Statistical significance was demonstrated through an unpaired two‐tailed Student's *t*‐test. Data are mean ± SEM; ^*^ *p* < 0.05; ^**^ *p* < 0.01.

Pathogenic CD8^+^ T cells are important in the development of SLE. However, the level and effector function of CD8^+^ T cells in peripheral blood and decidua of pSLE patients have not been examined. Here, we obtained peripheral blood and decidua from patients with pSLE and healthy pregnant controls (HPCs). We identified the percentage of CD3^+^ CD8^+^ T cells across CD45^+^ immune cells utilizing fluorescence‐activated cell sorting (FACS). The results showed that, compared with HPC, no significant differences were observed in the frequencies of CD3^+^ T cells throughout peripheral blood and decidua of patients with pSLE (Figure S1A,B). However, the levels of CD3^+^ CD8^+^ T cells in peripheral blood but not in decidua were significantly higher in pSLE patients, compared to HPC (Figure 1A,B).

Inflammatory cytokines IFN‐γ and TNF‐α are necessary for the maintenance as well as CD8^+^ T‐cell effector function. Therefore, we investigated the effector function of CD3^+^ CD8^+^ T cells through a comparison of the inflammatory cytokines IFN‐γ, TNF‐α, and IFN‐γ^+^ TNF‐α^+^ CD8^+^ T cells (a proxy for effector T‐cells persistence) generated by CD8^+^ T cells across the two groups. Compared to HPC, CD8^+^ T cells had significantly increased production of IFN‐γ, TNF‐α, and IFN‐γ^+^ TNF‐α^+^ CD8^+^ T cells in the peripheral blood of patients with pSLE (Figure 1A). In the decidua, only IFN‐γ produced by CD8^+^ T cells was significantly increased in patients with pSLE, compared to HPC, and the other categories were similar between groups (Figure 1B). Moreover, we used the surface marker to define the effector feature of CD8^+^ T cells (CD45RO^−^ CCR7^−^ CD95^+^ CD62L^−^). The result showed that, compared with HPC, CD8^+^ T cells in peripheral blood and decidua have much higher effector surface marker expression in pSLE (Figure S2A,B). Collectively, these findings indicate that the effector function of CD8^+^ T cells is significantly enhanced in pSLE patients.

### 2.2 EA levels are reduced in patients with pSLE

**FIGURE 2:**
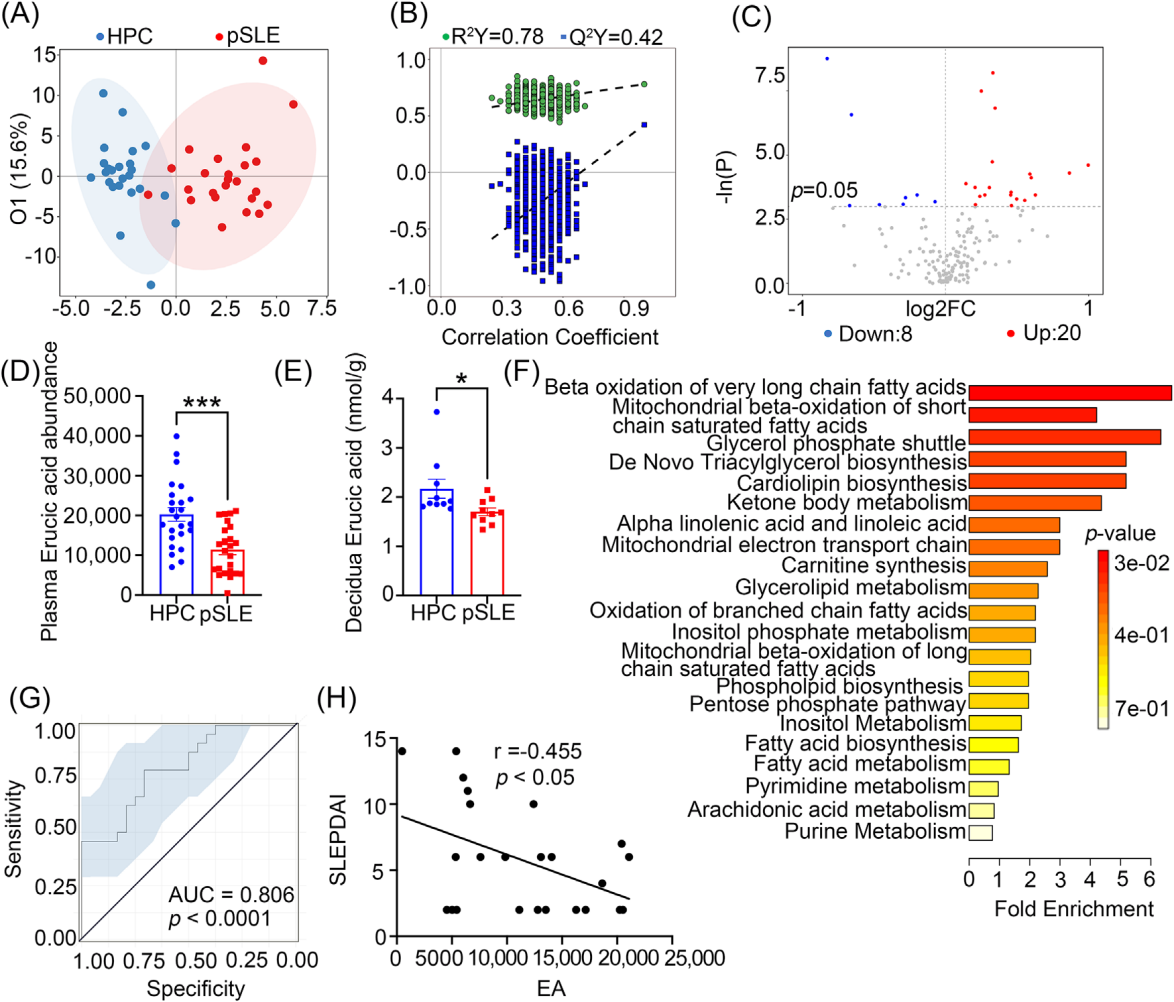
Erucic acid (EA) levels in plasma and decidua from patients with pSLE were significantly different, compared to HPC. (A, B) Orthogonal projections to latent structures discriminant analysis (OPLS‐DA) of the plasma metabolic profiles from patients with pSLE and HPC. The x‐axis represents between‐group differences, while the y‐axis denotes the difference between samples within the same group. The model of OPLS‐DA was verified using the permutation test. The y‐axis indicates the frequency of accuracy of the 200 models over the 200 displacement trials, while the dotted line denotes the accuracy of the OPLS‐DA, where Q2 indicates the predictive power of the model. (C) Volcano plot of differential metabolites in the plasma of patients with pSLE and HPC. Red dots indicate significantly upregulated metabolites; blue dots indicate significantly downregulated metabolites. (D) Untargeted metabolomics of plasma EA levels in pSLE patients (*n* = 24) and HPC (*n* = 24) contrasted utilizing an unpaired two‐tailed Student's *t*‐test. ^***^ *p* < 0.001. (E) Targeted metabolomics analysis was validated to compare EA levels in decidua between patients with pSLE (*n* = 10) and HPC (*n* = 10) using an unpaired two‐tailed Student's *t*‐test. ^*^ *p* < 0.05. (F) Metabolite set enrichment analysis shows changes in the top 20 metabolic pathways in plasma from patients with pSLE and HPC. (G) Receiver operating characteristic curve distinguishes patients with pSLE from HPC based on the plasma EA signature. (H) Spearman correlation analysis was used to compare EA levels in plasma and SLE Pregnancy Disease Activity Index (SLEPDAI) of patients with pSLE.

To explore metabolic changes in plasma composition between the HPC and patients with pSLE, we enrolled 24 patients with pSLE and 24 HPCs, all in the third trimester of pregnancy, who were hospitalized at Renji Hospital Department of Obstetrics (School of Medicine, Shanghai Jiao Tong University) between November 2020 and April 2021 into a clinical study. The pSLE and HPC groups had no significant difference in maternal age, gravidity, parity, body mass index (BMI) before pregnancy, and gestational week at which plasma samples were collected, but gestational weeks at the time of delivery and birth weight were lower, and preterm birth was increased in the pSLE group (Table 1). Plasma samples were analyzed using untargeted metabolomics, which showed that patients with pSLE clearly separated from HPC. Permutation testing was performed and indicated that the model was not over‐fitted (Figure 2A,B). Twenty‐eight metabolites were identified as present at different levels in the plasma of patients with pSLE, compared to HPC (Figure 2C). Among them, EA differed the most significantly, and plasma EA levels were reduced in pSLE patients, compared to HPC (Figure 2D). Further, targeted metabolomics confirmed that EA levels in decidua were significantly diminished in pSLE patients, compared to HPC (Figure 2E). Consistently, metabolite set enrichment analysis of all differential metabolites showed that β‐oxidation of very‐long‐chain FAs was significantly enriched (Figure 2F). Next, to explore the potential ability of EA to discriminate pSLE status, we generated receiver operating characteristic curves. This confirmed that the EA‐based classifier was able to accurately distinguish patients with pSLE from HPC (Figure 2G). Moreover, it was determined that EA level had a negative relationship with the SLE Pregnancy Disease Activity Index^21^ (SLEPDAI, Figure 2H). Collectively, these findings indicate that EA levels potentially have great diagnostic value for the prediction of disease severity in patients with pSLE.

**TABLE 1:** Baseline traits of healthy pregnant control (HPC) versus pregnancy complicated with systemic lupus erythematosus (pSLE) patients.

	HPC (*n* = 24)	pSLE (*n* = 24)	*p*‐value
Maternal age (years)	31.67 ± 3.56	30.21 ± 3.76	0.113
Gravidity (*N*)	1.83 ± 1.05	1.92 ± 1.28	0.982
Parity (*N*)	1.33 ± 0.48	1.13 ± 0.34	0.089
Pre‐pregnancy BMI (kg/m^2^)	20.70 ± 2.02	21.14 ± 2.18	0.472
Gestational week of plasma collection (weeks)	37.10 ± 3.25	35.94 ± 3.40	0.140
Pregnancy outcome			
Gestational weeks of delivery (weeks)	39.26 ± 1.23	36.49 ± 2.70	<0.001^***^
Preterm birth (*n*, %)	0	11(45.8)	<0.001^***^
Birth weight (*g*)	3305.00 ± 379.59	2707.29 ± 657.97	<0.001^***^
Fetal growth restriction (*n*, %)	0	3(12.5)	0.234

*Note*: Data are shown as mean ± SD or number (%).

*** *p* < 0.001.

### 2.3 EA inhibits the effector function in isolated CD8+ T cells of patients with pSLE

**FIGURE 3:**
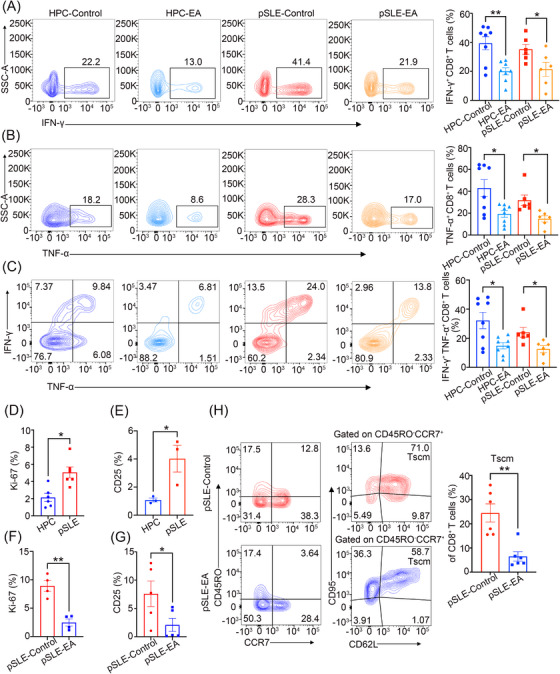
EA inhibits the effector function of CD8^+^ T cells. (A‐C) Naïve CD8^+^ T cells were obtained from peripheral blood mononuclear cells from patients with pSLE or HPC and activated using anti‐CD3/CD28 antibodies (25 μL/mL), with or without EA (0.5 mM) for 24 h. IFN‐γ, TNF‐α, and IFN‐γ^+^ TNF‐α^+^ CD8^+^ T cells produced by CD8^+^ T cells were analyzed by fluorescence‐activated cell sorting. (D) The expression of Ki‐67 between HPC (*n* = 6) and pSLE (*n* = 6). (E) The expression of CD25 between HPC (*n* = 3) and pSLE (*n* = 3). (F, G) Ki‐67 and CD25 of CD8^+^ T cells administered EA or not, for 24 h. (H) After anti‐CD3/CD28 stimulation, activated CD8^+^ T cells from patients with pSLE were maintained with human IL‐15 and treated with or without EA. Typical flow diagram and quantitative analysis are shown as CD45RO^−^CCR7^+^CD62L^+^CD95^+^ (Tscm subset; *n* = 6). Statistical significance was calculated utilizing an unpaired two‐tailed Student's *t*‐test. Data are mean ± SEM; ^*^ *p* < 0.05; ^**^ *p* < 0.01.

To examine the impact of EA on the effector function of CD8^+^ T cells, primary CD8^+^ T cells were obtained from PBMCs and treated with different levels of EA. The results showed that 0.5 mM EA for 24 h significantly inhibited the inflammatory cytokines IFN‐γ and TNF‐α produced by CD8^+^ T cells, as well as IFN‐γ^+^ TNF‐α^+^ CD8^+^ T cells (Figure S3A–C). No significant inhibitory effect was found after 0.5 mM EAtreated CD8^+^ T cells for 12 h (Figure S4A–C). EA treatment for 48 h inhibited inflammatory cytokine production by CD8^+^ T cells, but at this time, the cell activity is poor (Figure S4D–F). Therefore, we treatment CD8^+^ T cells from pSLE patients or HPC 0.5 mM EA for 24 h, which showed that EA‐treated CD8^+^ T cells produced significantly fewer IFN‐γ, TNF‐α, and IFN‐γ^+^ TNF‐α^+^ CD8^+^ T cells, compared to untreated cells (Figure 3A–C). These findings demonstrate that EA is able to inhibit the effector function of CD8^+^ T cells. Furthermore, we determined that the levels of the proliferation marker Ki‐67 and activation marker CD25 in peripheral blood CD8^+^ T cells of pSLE patients were elevated, compared to HPC (Figure 3D,E). However, Ki‐67 and CD25 levels in the CD8^+^ T cells of patients with pSLE were significantly decreased after EA treatment (Figure 3F,G).

To investigate whether EA can affect Tscm differentiation, we isolated naïve CD8^+^ T cells from patients with pSLE to generate CD8^+^ Tscm (CD45RO^−^ CCR7^+^ CD95^+^ CD62L^+^) cells. The results showed that, compared with untreated cells, EA treatment could significantly inhibit the differentiation of Tscm (Figure 3H). These results suggest that EA inhibit Tscm differentiation, thus reducing the production of pathogenic CD8^+^ T cells.

### 2.4 EA inhibits STAT3 hyperactivation in CD8^+^ T cells

**FIGURE 4:**
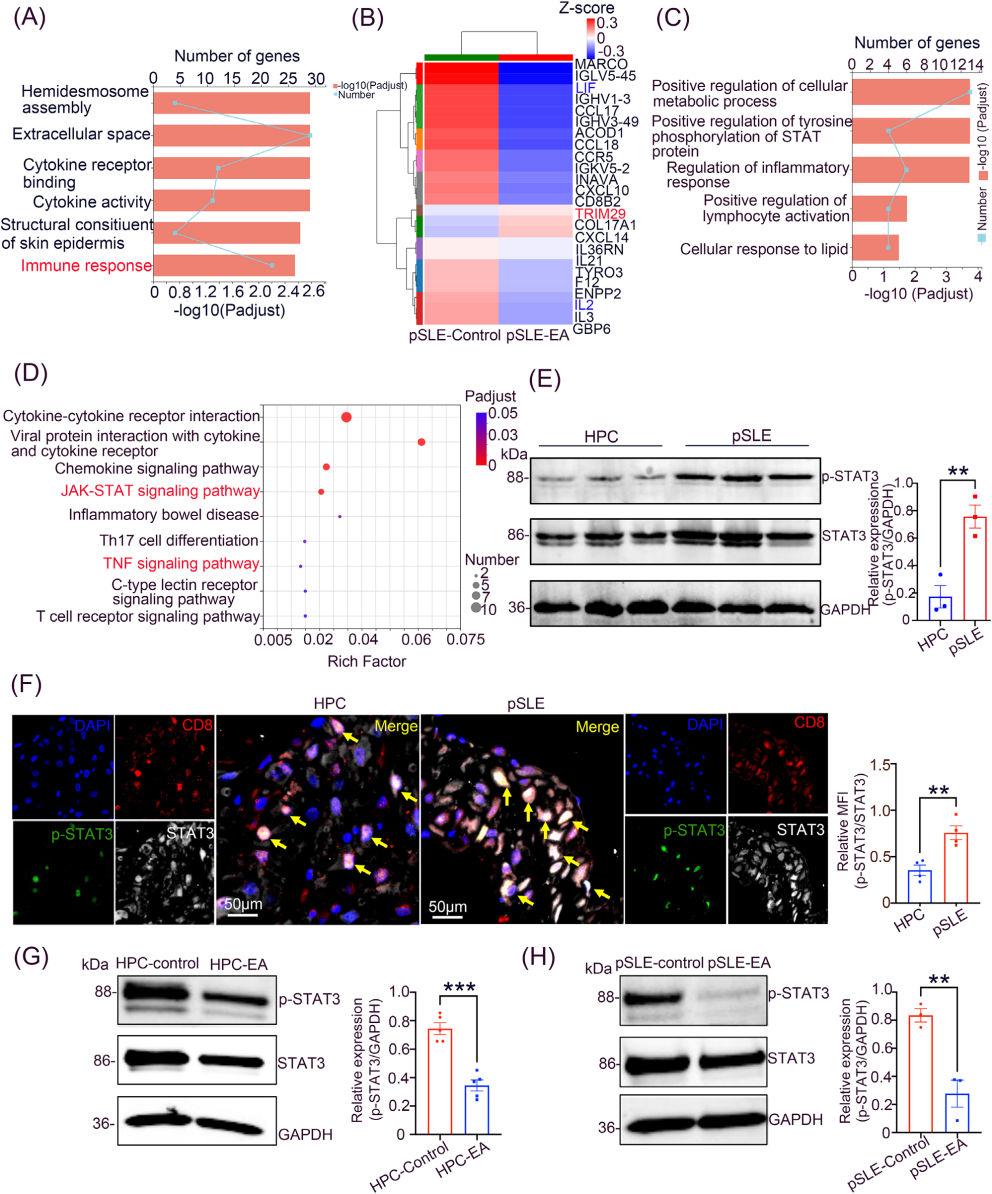
EA inhibits signal transducer and activator of transcription 3 (STAT3) hyperactivation in CD8^+^ T cells. (A) Gene ontology (GO) analysis of immune response target gene complements from CD8^+^ T cells from pSLE patients with or lacking EA treatment. (B) Heatmap of differential gene expression in the immune response target gene sets. (C, D) GO and Kyoto Encyclopedia of Genes and Genomes (KEGG) enrichment analysis of immune response target gene sets. (E) Phosphorylated (p)‐STAT3 (p‐STAT3) protein levels in decidua from patients with pSLE (*n* = 3) and HPC (*n* = 3). (F) Mean fluorescence intensity of p‐STAT3 on CD8^+^ T cells in decidua from patients with pSLE (*n* = 4) and HPC (*n* = 4) were determined and normalized by STAT3, scale bar = 50 μm, yellow arrows indicate cells that are co‐located by CD8, p‐STAT3, and STAT3. (G, H) Protein levels of p‐STAT3 in CD8^+^ T cells obtained from pSLE patients or HPC‐treated or lacking EA. Experiments were conducted in biological triplicate. Statistical significance was computed using unpaired two‐tailed Student's *t*‐test. Data are mean ± SEM; ^*^ *p* < 0.05; ^**^ *p* < 0.01.

RNA‐seq was conducted to investigate the potential mechanism through which EA limits the effector function of CD8^+^ T cells. Specifically, CD8^+^ T cells from pSLE patients were separated using magnetic beads, incubated with EA for 24 h, and the cells were then subjected to RNA‐seq and analysis. We observed significantly different gene expression patterns of CD8^+^ T cells after EA treatment, compared to untreated cells, and gene ontology (GO) enrichment analysis showed that EA treatment enriched genes associated with immune response (Figure 4A). We therefore created a target gene set for immune response and performed cluster analysis to determine the levels of differentially expressed genes linked to effector function of CD8^+^ T cells. We identified significantly reduced expression of leukemia inhibitory factor (*LIF*), a gene related to the TNF‐activation signaling pathway, and interleukin 2 (*IL2*), a gene related to T‐cell growth and differentiation, whereas tripartite motif‐containing protein 29 (*TRIM29*), a gene that negatively regulates protein entry into the nucleus, was significantly increased in EA‐treated CD8^+^ T cells (Figure 4B). GO analysis also showed that the gene functions within the immune response sets were mainly related to intracellular metabolism, STAT protein phosphorylation, and the inflammatory response (Figure 4C), and Kyoto Encyclopedia of Genes and Genomes (KEGG) analysis demonstrated that the TNF signaling pathway was enriched significantly throughout the target gene set for immune response (Figure 4D). Together, our findings indicate that EA influences functional alterations in CD8^+^ T cells over the course of an immune response that modulates downstream inflammatory responses and intracellular metabolism changes.

KEGG analysis exhibited that the Janus Kinase (JAK)/STAT signaling pathway was significantly enriched in CD8^+^ T cells following EA treatment (Figure 4D). Immunoblotting of protein extracts from decidua demonstrated that phosphorylated (p)‐STAT3 was significantly elevated in pSLE patients, compared to HPC (Figure 4E). To examine the impact of p‐STAT3 expression on CD8^+^ T cells between patients with pSLE and HPC, we performed multicolor immunofluorescence staining on decidua, which demonstrated that the mean fluorescence intensity of p‐STAT3 on CD8^+^ T cells was significantly increased in patients with pSLE relative to HPC (Figure 4F). Importantly, EA treatment significantly reduced the expression of p‐STAT3 in CD8^+^ T cells arising from the peripheral blood of pSLE patients or HPC (Figure 4G,H). Furthermore, upon treatment of CD8^+^ T cells from HPC or patients with pSLE with the STAT3 inhibitor Stattic, the levels of IFN‐γ, TNF‐α, and IFN‐γ^+^ TNF‐α^+^ CD8^+^ T cells were significantly diminished (Figure S5A–C). Our results demonstrate that EA governs the effector function of CD8^+^ T cells through the inhibition of STAT3 hyperactivation.

### 2.5 EA enhances lipid peroxidation and ferroptosis in CD8^+^ T cells

**FIGURE 5:**
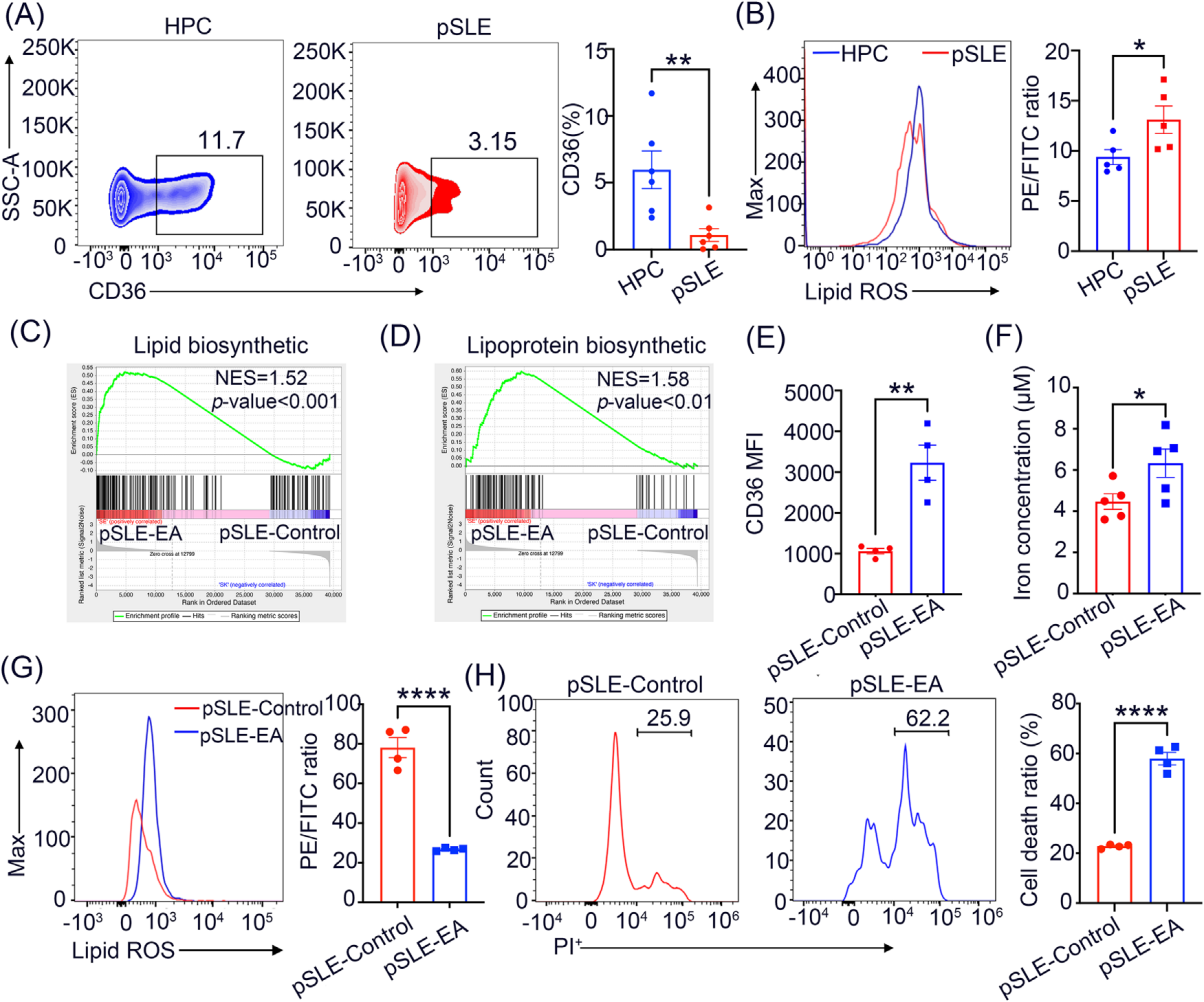
EA p lipid peroxidation and ferroptosis in CD8^+^ T cells. (A) Levels of CD36 from peripheral blood CD8^+^ T cells in pSLE patients (*n* = 6) and HPC (*n* = 6). (B) Lipid peroxidation in peripheral blood CD8^+^ T cells from patients with pSLE (*n* = 5) and HPC (*n* = 5). (C, D) Gene set enrichment analysis derived from RNA‐sequencing data was utilized to assess changes in lipid metabolism‐related pathways in CD8^+^ T cells in the presence or absence of EA treatment. (E) Expression of CD36 on CD8^+^ T cells obtained from pSLE patients after EA treatment for 24 h. (F) Iron concentration. (G) Lipid peroxidation. (H) Cell death ratio (propidium iodide^+^ [PI^+^] %) in CD8^+^ T cells following EA treatment for 24 h. Statistical significance was calculated utilizing an unpaired two‐tailed Student's *t*‐test. Data are mean ± SEM; ^*^ *p* < 0.05; ^****^ *p* < 0.0001.

We next examined the molecular mechanism by which EA controls the CD8^+^ T‐cell effector function in pSLE patients. FACS analysis showed that, compared to HPC, expression of CD36 on CD8^+^ T cells arising from peripheral blood was significantly reduced in pSLE patients (Figure 5A). Accordingly, we employed a lipid peroxidation assay for the detection of cellular lipid reactive oxygen species in CD8^+^ T cells obtained from peripheral blood. The findings indicated that the level of lipid peroxidation was reduced in CD8^+^ T cells from pSLE patients relative to HPC as indicated by a higher PE/FITC ratio (Figure 5B). Gene set enrichment analysis on CD8^+^ T cells from pSLE patients demonstrated that lipid biosynthetic and lipoprotein biosynthetic pathways were greatly enriched by EA treatment (Figure 5C,D). Further, the expression of CD36, cellular iron concentration, and lipid peroxidation, alongside the cell death ratio of CD8^+^ T cells were significantly upregulated after EA treatment (Figure 5E–H). These findings indicate that EA is linked to ferroptosis in CD8^+^ T cells.

### 2.6 EA promotes ferroptosis in CD8^+^ T cells resulting in reduced effector function

**FIGURE 6:**
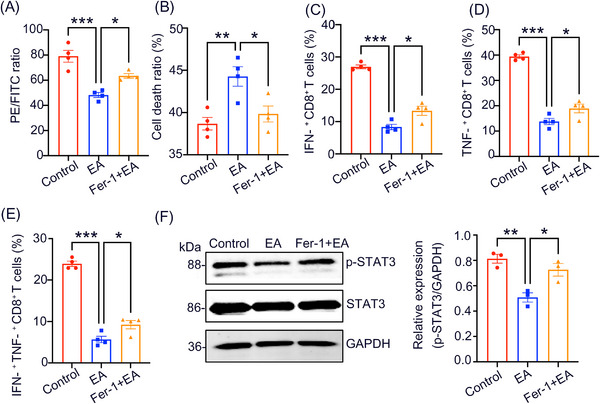
EA enhances ferroptosis in CD8^+^ T cells is responsible for reduced CD8^+^ T cell effector function. (A–E) CD8^+^ T cells were obtained and stimulated using anti‐CD3/CD28 antibodies and treated with EA (0.5 mM) and Fer‐1 (10 μM) for 24 h. CD8^+^ T cells were examined for lipid peroxidation, cell death ratio (PI^+^ %), and the production of IFN‐γ, TNF‐α, and IFN‐γ^+^ TNF‐α^+^ CD8^+^ T cells. (F) Cultured CD8^+^ T cells obtained from pSLE patients were treated with EA or Fer‐1 for 24 h and then p‐STAT3 protein was detected by immunoblotting. One‐way analysis of variance (ANOVA) was conducted across three groups. Data are mean ± SEM; ^*^ *p* < 0.05; ^***^ *p* < 0.001.

Ferroptosis is a cell death form caused by iron accumulation alongside lipid peroxidation. To determine whether EA‐promoted ferroptosis was required for the decreased effector function of CD8^+^ T cells in patients with pSLE, CD8^+^ T cells from pSLE patients were obtained, treated with EA and the ferroptosis inhibitor (Fer‐1), and stimulated using anti‐CD3/CD28 antibodies in vitro. We then analyzed lipid peroxidation, cell death, and the production of IFN‐γ, TNF‐α, IFN‐γ^+^ TNF‐α^+^ CD8^+^ T cells in treated CD8^+^ T cells. The results showed that Fer‐1 partially inhibited the EA‐induced increase in lipid peroxidation and cell death ratio (Figure 6A,B) and restored the EA‐depleted IFN‐γ, TNF‐α, and IFN‐γ^+^ TNF‐α^+^ CD8^+^ T cells (Figure 6C–E). Fer‐1 reversed EA‐mediated inhibition of p‐STAT3 expression in CD8^+^ T cells (Figure 6F). These findings demonstrate that EA is responsible for inhibiting the effector function of CD8^+^ T cells by reducing STAT3 phosphorylation and promoting ferroptosis.

### 2.7 EA supplementation inhibits the effector function of CD8^+^ T cells in pregnant MRL/lpr mice

**FIGURE 7:**
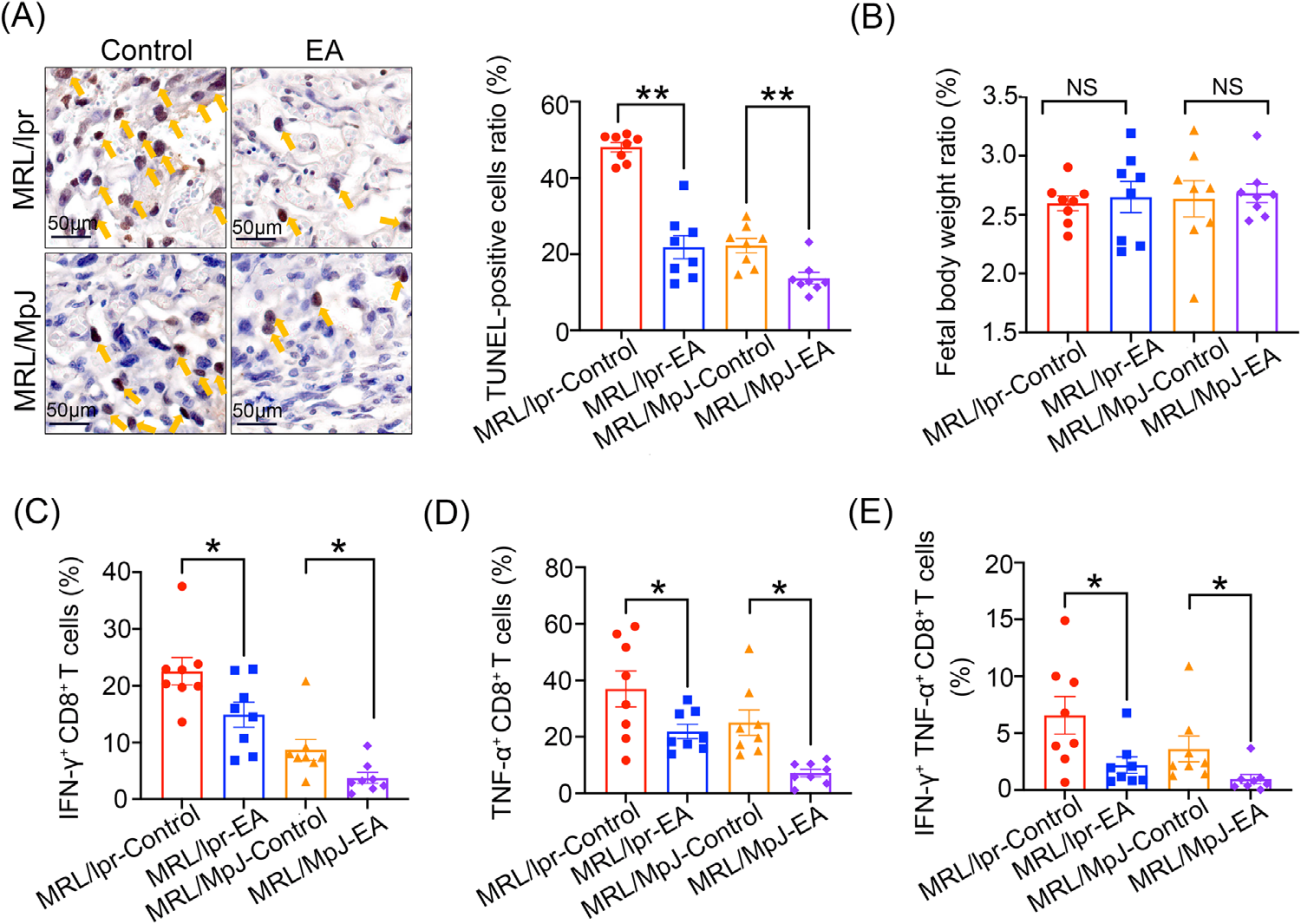
EA supplementation prevents the effector function of uterine CD8^+^ T cells throughout pregnant MRL/lpr mice. (A) Mice were given EA by oral gavage on day 0.5, 5.5, 10.5, and 15.5 of gestation. Transferase dUTP nick end labeling (TUNEL) staining in the placenta of MRL/lpr‐Control group (*n* = 8), MRL/lpr‐EA group (*n* = 8), MRL/MpJ‐Control group (*n* = 8), and MRL/MpJ‐EA group (*n* = 8), scale bar = 50 μm, the orange arrows indicate apoptotic or necrosis cells. (B) Fetal body weight ratio among four group. (C–E) Flow cytometric analysis of the proportion of IFN‐γ, TNF‐α, and IFN‐γ^+^ TNF‐α^+^ CD8^+^ T cells generated by CD8^+^ T cells in the uterus of MRL/lpr‐Control group (*n* = 8), MRL/lpr‐EA group (*n* = 8), MRL/MpJ‐Control group (*n* = 8), and MRL/MpJ‐EA group (*n* = 8) with or without EA treatment. Statistical significance was assessed using unpaired two‐tailed Student's *t*‐test. Data are mean ± SEM; ^*^ *p* < 0.05; ^**^ *p* < 0.01.

EA supplementation has been demonstrated to markedly enhance prognosis in virus‐infected mice through the reduction of CD8^+^ T‐cell aggregation.^16^ Nevertheless, the function of EA in pregnant MRL/lpr mice—an autoimmune mouse model of SLE—has not been examined. Pregnant MRL/lpr mice and control MRL/MpJ mice were separated into groups receiving EA or vehicle control. Mouse placentas were characterized by apoptotic or necrotic cells. TUNEL staining was utilized to assess the effects of EA on the placenta of MRL/lpr mice. The findings demonstrated a higher severity of placental injury in MRL/lpr mice, compared to in MRL/MpJ mice. Furthermore, EA treatment alleviated apoptosis and necrosis in the placenta of MRL/lpr mice relative to untreated mice (Figure 7A). No significant alterations in fetal body weight ratio were observed between pregnant mice with MRL/lpr with or lacking EA treatment (Figure 7B). We observed that EA did not cause pathological damage to the heart and liver of pregnant MRL/lpr and MRL/MpJ mice (Figure S6).

To further examine the impact of EA on immune cell function in the uterus of pregnant MRL/lpr mice, we utilized FACS to identify CD3^+^ CD8^+^ T‐cell effector function. The results showed that pregnant MRL/lpr mice received EA supplementation had significantly decreased production of IFN‐γ, TNF‐α, and IFN‐γ^+^ TNF‐α^+^ CD8^+^ T cells by CD8^+^ T cells in the pregnant uterus, compared to untreated mice (Figure 7C–E). Collectively, these findings demonstrate that EA improves placental damage in MRL/lpr mice through the inhibition of effector function in CD8^+^ T cells.

## 3 DISCUSSION

Maternal–fetal outcomes in patient with pSLE have greatly improved in recent decades, but pregnancy is still associated with a high level of complications for women with SLE. It is well established that abnormal CD8^+^ T cell functioning is intimately tied to the progression of autoimmune and inflammatory reactions in SLE. However, the regulation underlying the abnormal activation of pathogenic CD8^+^ T cells in pSLE patients remains undefined. In our study, we determined that increased effector function in CD8^+^ T cells was accompanied by lowered EA levels in pSLE patient, suggesting that CD8^+^ T cells alongside EA disturbances play important roles this disease. We also uncovered that EA could inhibit the effector function of CD8^+^ T cells, providing a putative therapeutic strategy for pSLE patients.

The consistent role of CD8^+^ T cells underscores the complete pathogenesis of SLE and has attracted substantial attention.^8^ CD8^+^ T cells can enhance the appearance and development of autoimmune diseases through dysregulation of inflammatory cytokines IFN‐γ and TNF‐α production.^22^ IFN‐γ and TNF‐α are required for CD8^+^ T‐cell accrual and effector function maintenance.^23^ Study have shown that increased IFN‐γ production leads to increased severity of lupus nephritis.^24^ Anti‐IFN‐γ treatment substantially reduced the progression of glomerulonephritis in lupus mice.^25^ The level of TNF‐α is very low under normal circumstances. In patients with SLE and other autoimmune disease, the level of TNF‐α is significantly increased, which destroys the immune balance, promotes the occurrence of inflammatory response, and intimately leads to pathological damage of tissue and organs.^26^ TNF blocking therapy may enable long‐term remission in lupus nephritis patients.^27^ The inflammatory cytokine IFN‐γ and TNF‐α generated by CD8^+^ T cells were significantly enhanced in pathological pregnancies including recurrent abortion or preeclampsia.^28^ Examination of the effector function of CD8^+^ T cells in pSLE patients highlighted that IFN‐γ produced by CD8^+^ T cells increased substantially in peripheral blood and decidua of patients with pSLE. TNF‐α levels generated by CD8^+^ T cells grew significantly in the peripheral blood of patients with pSLE. While TNF‐α production by CD8^+^ T cells did not differ significantly in decidua, the proportion of effector CD8^+^ T cells in peripheral blood and decidua was elevated significantly in pSLE patients. Alongside previous reports, our findings indicate that improved effector function in CD8^+^ T cells may be involved in the occurrence of placental injury in patients with pSLE.

EA was reported to inhibit the inflammatory response by inhibiting proinflammatory mediators and recruitment of CD8^+^ T cells.^16^ We identified for the first time that EA levels were diminished significantly in pSLE patients. Correlation analysis demonstrated that EA levels exhibited a negative relationship with disease severity in patients with pSLE. Moreover, plasma EA levels were also significantly negatively correlated with the presence of adverse pregnancies in pSLE patients (*r* = −0.415, *p*‐value = 0.044). This finding indicates that reduced EA levels in patients with pSLE may have a diagnostic benefit in the prediction of adverse pregnancy outcomes in patients with pSLE. To date, a limited number of investigations have examined EA's impact on the function of CD8^+^ T cells. In our study, we found that EA could significantly reduce IFN‐γ and TNF‐α produced by CD8^+^ T cells, implying that EA could limit the effector function of CD8^+^ T cells.

JAK/STAT3 pathway is critical for resistance to infection and maintenance of immune tolerance, and dysregulating this pathway may lead to autoimmune disease.^29^, ^30^, ^31^ Tofacitinib (inhibitor) can effectively inhibit the effector function of CD8^+^ T cells, enhancing renal function in lupus mice.^32^ STAT3 governs the differentiation and effector function of CD8^+^ T cells throughout cancer and autoimmune diseases and has received widespread attention.^33^, ^34^ Previous investigations have demonstrated that increased STAT3 or decreased lipid peroxidation improves the effector and longevity function of CD8^+^ T cells.^18^ Specially, it has been reported that inhibition of STAT3 can result in ferroptosis through the promotion of lipid peroxidation and ferrous iron (Fe^2+^) accumulation.^35^ In our current study, we identified that EA could significantly inhibit STAT3 phosphorylation, promoting lipid peroxidation and iron accumulation in CD8^+^ T cells. When we supplement ferroptosis inhibitor, it can partially reverse this phenomenon. Our current finding suggests that EA inhibits effector function in CD8^+^ T‐cells effector function by inhibiting STAT3 phosphorylation and promoting ferroptosis.

MRL/lpr mouse is a relatively mature spontaneous lupus mouse model. The main feature of this mouse is the excessive proliferation of T cells, accompanied by the enlargement of lymph nodes, erosive arthritis, and the production of autoantibodies. They are prone to miscarriage and low birth weight after pregnancy.^37^ As the important pro‐inflammatory cytokines, IFN‐γ and TNF‐α contribute to the progression of autoimmune diseases,^38^, ^39^ overproduction of inflammatory cytokines can lead to pathological damage of the placenta, which can lead to adverse pregnancy outcomes.^40^ Our findings reveal that EA treatment inhibited IFN‐γ and TNF‐α production by CD8^+^ T cells in the uterus of pregnant lupus mice and improved the placental damage in lupus mice, thereby revealing the putative therapeutic benefit of EA in pSLE disease.

It was reported that EA has both toxic and beneficial neuropharmacological effects according to its concentration. Previous studies showed that EA can result in myocardial lipidosis, heart lesions, and hepatic steatosis in animals, while a combination of EA and oleic acid, commonly utilized as a dietary treatment for the management of adrenoleukodystrophy, is not linked to cardiotoxicity.^41^ Another study showed that extended administration of an experimental high‐EA diet to rats or hamsters did not cause marked histopathological changes throughout pregnancy to the liver, heart, kidneys, or adrenals.^42^ We also supplemented the hematoxylin‐eosin (H&E) staining of the hearts and livers of pregnant lupus mice and their control group after EA treatment. The results showed that EA did not cause heart and liver damage in pregnant mice (Figure S6). The safety evaluation of EA in infant formula shows that EA in edible oils was much lower than 4 g/kg. Therefore, these oils would be well‐suited to prepare homemade infant and childhood food.^43^, ^44^ Notably, data from the International Agency for Research on Cancer suggest that EA contained in the Chinese diet can significantly reduce the occurrence of infant brain tumors, compared to the Western diet.^45^ To sum up, there is not enough evidence‐based medical evidence to prove the adverse effects of EA, and the edible oils we can get from the market are relatively safe for infants. Further reliable and timely evidence‐based data on EA are needed.

This study had important limitations that must be considered when interpreting and applying the data. First, in the clinical study, although maternal age, gestational week, gravidity, and parity were similar between patients with pSLE and HPC, it is possible that those medications taken by patients with pSLE influenced metabolomic profiles. However, almost all the SLE women who have a successful pregnancy to term would take these medications even before the pregnancy is confirmed. In our study, the dosage of prednisone ranged from 0 to 40 mg/d, and hydroxychloroquine(HCQ) ranged from 0.1 to 0.4 g/d as shown in Table S2. Regarding the effect of medication on the effect of EA, we conducted a Spearman correlation analysis on the dosage of prednisone and the plasma EA levels and found no correlation between them (*r* = 0.297, *p*‐value = 0.158), no correlation was identified between HCQ and EA (*r* = 0.189, *p*‐value = 0.375), which indirectly reflected that the disease of pSLE was a factor affecting EA level. Meanwhile, our study showed that prednisone and HCQ combination cannot completely inhibit the inflammatory response of CD8^+^ T cells in patients with pSLE. Thus, we proposed that EA adjuvant therapy might bring benefits to patients with pSLE. Second, ferroptosis is a complicated process in vivo, which is triggered by multiple factors and mechanisms.^36^ Our study established that the levels of CD36 and lipid peroxidation in CD8^+^ T cells in pSLE patients were significantly reduced, compared to those in HPC. EA could enhance the lipid peroxidation and iron collection in CD8^+^ T cells. Other potential mechanisms by which EA mediates ferroptosis of CD8^+^ T cells remain to be further investigated. Finally, EA levels change during pregnancy, and the effect of EA on immune status may develop slowly, beginning during early pregnancy. While the trend for EA levels in decidua was consistent with those in peripheral blood, suggesting blood EA levels may be a useful predictive peripheral biomarker, our study did not sufficiently study EA levels in decidua during early pSLE. The proportion of immune cells and metabolites levels throughout pregnancy are dynamic, and thus indicators used in early pregnancy are not appropriately applied to late pregnancy. Most importantly, the occurrence of premature delivery and preeclampsia in patients with pSLE was significantly increased, compared to that of HPC, and these diseases mainly occur during late pregnancy. Therefore, metabolomic changes in late pSLE may provide obstetricians with more accurate warning indicators that can be acted upon to improve maternal and child safety. However, if future studies can determine clinically meaningful EA changes during early pSLE, it could support early intervention and potentially have a greater impact on maternal‐fetal outcomes.

## 4 CONCLUSION

Our study recognizes EA as a dietary nutrient capable of inhibiting the effector function of CD8^+^ T cells, suggesting that EA supplementation might provide a therapeutic approach to treat pSLE disease.

## 5 MATERIALS AND METHODS

### 5.1 Study subjects and sample collection

The study received approval from the Ethics Committee of Renji Hospital (School of Medicine, Shanghai Jiao Tong University). All participants offered written informed consent and the investigation was performed in alignment with the Helsinki Declaration. Patients with pSLE (*n* = 90) or healthy control patients (HPC) (*n* = 62) were recruited among inpatients at the Renji Hospital Department of Obstetrics (School of Medicine, Shanghai Jiao Tong University). The clinical attributes of all participants are outlined in Table S1. pSLE patients attained the 2012 Systemic Lupus International Collaborating Clinics criteria for SLE. Exclusion criteria included multiple pregnancies, hypertension and diabetes before delivery, and infectious disease (including acute infection and chronic infection or inflammation of the immune system). Participants offered written informed consent pertaining to cesarean section delivery. Naïve CD8^+^ T cells were obtained from the blood of pSLE patients or HPC, and decidua tissue collection was completed within 30 min of delivery.

### 5.2 Cell preparation and culture

We obtained peripheral blood mononuclear cells (PBMCs) from pSLE patients or HPC through density gradient separation. Naïve CD8^+^ T cells were isolated from PBMCs utilizing a human naïve CD8^+^ T‐cell isolation kit (#17853 STEMCELL Technologies) according to the manufacture instructions. Then, 2−3 × 10^5^ cells were stimulated using human T‐cell anti‐CD3/CD28 activator (#10971 STEMCELL Technologies) in X‐vivo (#04‐418Q, Lonza) complete medium for 24 h, and activated CD8^+^ T cells were either given EA or not. A total of 25 ng/mL recombinant human interleukin (IL)‐15 (#200‐15, Peprotech) was included with the activated CD8^+^ T cells to prompt the Tscm differentiation states.

### 5.3 Flow cytometric analysis

The following antibodies targeting human proteins were employed for FACS: anti‐CD45 (PRID: AB_2563466), anti‐CD3 (PRID: AB_314060), anti‐CD8 (PRID: AB_2869551), anti‐CD8 (PRID: AB_314126), anti‐IFN‐γ (PRID: AB_28011024), anti‐TNF‐α (PRID: AB_2565858), anti‐CD25 (PRID: AB_314274), anti‐ki‐67 (PRID: AB_2562872), anti‐CD45RO (PRID: AB_314421), anti‐CCR7 (PRID: AB_10916121), anti‐CD62L (PRID: AB_314470), anti‐CD95 (PRID: AB_314544), anti‐CD36 (PRID: AB_1575025), and propidium iodide (#421301, Biolegend). The following antibodies against mouse proteins were utilized: anti‐CD45 (PRID: AB_893340), anti‐CD3 (PRID: AB_312660), anti‐CD8 (PRID: AB_312747), anti‐CD44 (PRID: AB_2562451), anti‐CD62L (PRID: AB_313102), anti‐IFN‐γ (PRID: AB_2734493), and anti‐TNF‐α (PRID: AB_2565951).

Single‐cell suspensions were stained with an appropriate Fc receptor blocker prior to surface staining. Intracellular staining was first stimulated by Leukocyte Activation Cocktail with BD GolgiPlug (PRID: AB_2868893) for 4−6 h to induce the inflammatory cytokines. Next, a Fixation/Permeabilization kit (PRID: AB_2869424) was used for further staining. Lipid peroxidation was detected using a BODIPY 581/591 Lipid Peroxidation Sensor (10uM, #RM02821, ABclonal) for 30 min in a culture medium prior to staining. Following incubation, cells were harvested and examined through flow cytometry within 2 h of staining. Cells were obtained and the different phenotypes were examined using LSR Fortessa (BD Biosciences). Flow cytometry data were assessed using FlowJo software (BD Biosciences).

### 5.4 Cellular iron staining

Intracellular iron of CD8^+^ T cells was detected using the Intracellular Iron Colorimetric Assay Kit (#E1042, APPLYGEN), which employs a colorimetric detection method. Specifically, cells were acquired and washed twice with cold phosphate buffered saline (PBS), lysed for 2 h on a 4°C shaker, and incubated alongside potassium permanganate for 1 h at 60°C. Subsequently, the iron detector reagent was added for 30 min. Iron absorbance at 550 nm was examined and concentration was determined by generating a standard curve.

### 5.5 Metabolomics

Untargeted metabolomics profiling was conducted employing an XploreMET platform (Metabo‐Profile). The project was performed under the guidance of the Quality Management System. For the determination of metabolites in plasma, the plasma samples were melted on ice and centrifuged, and 50 μL of liquid was combined with 10 μL of internal standard. Thereafter, 175 μL of pre‐cooled methanol/chloroform (v/v = 3/1) was included and samples were spun at 14,000 g for 20 min. Next, 200 μL of supernatant was relocated to an automatic sample injection bottle, and the rest was used as a quality control sample. The metabolite extract was removed by centrifugation and freeze‐dried to a dry powder.

Targeted metabolomics profiling was performed by Metabo‐Profile Biotechnology. Decidua tissue (∼10 mg) was collected into a microcentrifuge tube, and the samples were mixed with 10 pre‐cooled oxidized magnetic beads alongside 20 μL of deionized water. We then homogenized the samples for 3 min, and 120 μL containing internal standard methanol was added to obtain metabolites for 3 min. Samples were spun at 18,000 g for 20 min and moved to a 96‐well plate. The procedures were performed using a Biomek 4000 workstation: Derivatization, reconstitution and centrifugation, and transfer of supernatant with internal standards from each well to a new 96‐well plate that was sealed for liquid chromatography‐mass spectrometry (LC−MS) analysis. Both untargeted and targeted metabolomics analysis reports were prepared by Metabo‐Profile Biotechnology (Shanghai) Co., Ltd.

### 5.6 RNA‐sequencing analysis (RNA‐seq)

CD8^+^ T cells from the peripheral blood of patients with pSLE were cultured with EA or not for 24 h. Complete RNA was obtained from the CD8^+^ T cells using TRIzol Reagent based on the standard protocol. RNA quality was assessed through the use of a 2100 Bioanalyser (Agilent) and quantified utilizing an ND‐2000 spectrophotometer (NanoDrop Technologies). The RNA‐seq transcriptome library was compiled and qualified, and the RNA‐seq library was sequenced using an Illumina HiSeq xten/NovaSeq 6000 sequencer (2 × 150 bp read length). Differential gene expression examination and functional enrichment were conducted through the online Majorbio Cloud Platform (www. Majorbio.com).

### 5.7 Immunoblotting and multicolor immunofluorescence staining

Decidua tissue and cultured CD8^+^ T‐cell proteins were collected and incubated within radioimmunoprecipitation assay lysis buffer (#P0013B, Beyotime Biotechnology) alongside protease and phosphatase inhibitors for 30 min on ice. Total protein was acquired by centrifugation at 12,000 rpm for 20 min at 4°C and quantified utilizing a bicinchoninic acid assay kit (#P0012S, Beyotime Biotechnology). The protein samples were pipetted into 10% sodium dodecyl sulphate‐polyacrylamide gel electrophoresis (SDS−PAGE)gels for electrophoresis and transmitted to polyvinylidene fluoride (PVDF) membranes. The primary antibodies used were anti‐p‐STAT3 (#ab76315, Abcam), anti‐STAT3(#4904T, Cell Signaling Technologies), and anti‐GAPDH (#ARG10112, Arigo). Signals were distinguished utilizing an Odyssey Infrared Imaging System (LI‐COR Biosciences) or chemical luminescence (Bio‐Rad), and the blots were qualified using Image J.

Formalin‐fixed, paraffin‐embedded decidua was used for multicolor immunofluorescence. Briefly, decidua was placed in an oven at 60°C for 2 h, rehydrated, and repaired with sodium citrate antigen in a microwave oven for 30 min. After blockage the decidua tissue with bovine serum albumin (BSA) for 1 h, and incubation with primary antibody anti‐CD8, anti‐p‐STAT3, and anti‐STAT3, overnight at 4°C. After rinsing three times with PBS, decidua was incubated alongside three diverse fluorescently conjugated secondary antibodies at room temperature for 1 h and stained using 4, 6‐diamidino‐e‐phenylindole (#ab104139, Abcam) to detect cell nuclei.

### 5.8 Animal experimentation

Animal investigations were conducted according to the guidelines of the Institutional Animal Care and Use Committee of Renji Hospital (School of Medicine, Shanghai Jiao Tong University), and the animal protocols were authorized by the same committee. This study followed the Animal Research: Reporting of in vivo experiments (ARRIVE) guidelines for in vivo studies on animals. MRL/lpr and MRL/MpJ (female, 8−9 weeks old) were purchased from Shanghai Slack Laboratory. MRL/lpr and MRL/MpJ mice were randomly housed overnight with corresponding male mice at 2:1 ratio and assigned to the experiment or control group 1 day following vaginal spermatozoa was observed and gestational day (GD) 0.5 was verified. The mice in the experiment group were given 75 mg/kg EA (#E3385‐1G, Merck) by gavage on GD 0.5, 5.5, 10.5, and 15.5. The dosage of EA was referred to the method of Liang et al.^16^ we also used different concentrations of EA (50, 75, 150 mg/kg) and administration times (once every 2 days and every 4 days by oral gavage, and the results showed that only when we give 75 mg/kg once 4 days, the pregnant MRL/lpr mice were in a good state. Abdominal splenomegaly as well as mesenteric lymph node enlargement was significantly lowered in pregnant MRL/lpr mice, compared to untreated mice. The control group received an equivalent volume of PBS (#G4202, Servicebio) at the same time points. The cohort sizes were as follows: MRL/lpr‐Control group, eight; MRL/lpr‐EA group, eight; MRL/MpJ‐Control group, eight; and MRL/MpJ‐EA group, eight. Mouse plasma was collected at day 17.5 of gestation, and anti‐dsDNA in plasma was detected by Elisa (#5120, Alpha Diagnostic Intl). We found that the level of anti‐dsDNA in MRL/lpr was significantly higher than MRL/MpJ mice (MRL/lpr vs. MRL/MpJ, 4.45U/mL × 10^6^ ± 2.0 vs. 0.13U/mL ×10^6^ ± 0.03, *p*‐value < 0.05). On GD 17.5, pregnant mice were sacrificed through cervical dislocation following anesthesia with pentobarbital (0.3%, 1 mL/100 g, #1030001‐25MG, Merck). We used the fetal weight ratio to accurately compare fetal weight between different groups, that is, the fetal weight ratio is equal to the fetal weight divided by the mother's weight at the time of vaginal spermatozoa was confirmed. The live, heart, placenta and uterus of pregnant mice were collected. Mouse uteri were separated into single‐cell suspensions for flow cytometric analysis.

### 5.9 Histological review

H&E staining was utilized for the detection of anatomical traits of the mouse heart and liver. Two pathologists with perinatal pathology experience reviewed H&E‐stained sections to confirm the diagnosis. The specific experimental procedures were as follows: paraffin removal, rehydration, hematoxylin staining for 5 min, eosin staining for 1 min, dehydration and air drying, and neutral resin sealing.

### 5.10 Staining through terminal deoxynucleotidyl transferase dUTP nick end labeling (TUNEL)

TUNEL staining was utilized for the detection of necrosis and apoptosis in mice placenta. Staining proceeded using the manufacturer's instructions. The determination of the positive rate was based on dividing the number of TUNEL‐positive cells by the total number of cells in the same area.

### 5.11 Statistical investigation

Data analysis was conducted utilizing IBM SPSS Statistics (Version 25) as well as the free online Majorbio I‐Sanger Cloud Platform (www.i‐sanger.com). To contrast parameters between two groups, if the data were normally distributed, a two‐tailed unpaired Student's *t*‐test was used with similar variances, and a two‐tailed unpaired Student's *t*‐test with Welch's correction was performed when the variances were varied. For the evaluation of differences between the three groups, a one‐way analysis of variance (ANOVA) was utilized. A Mann–Whitney U test was employed for continuous variables with non‐normal distribution, and a Chi‐square test or Fisher exact test was utilized for categorical variables. Spearman correlation analysis was employed to contrast EA levels in plasma and SLEPDAI of patients with pSLE. Experiments were conducted at least in triplicate. Figures were generated utilizing Prism software (Version 9.0). *p*‐value < 0.05 was statistically significant.

## AUTHOR CONTRIBUTIONS

Y.L.C. wrote the first draft of the study protocol. Y.L.C. and M.J. carried out experiments. M.J. provided comments and final approval. Y.W. and S.H.L. were responsible for clinical sample collection. Q.F., J.Y.W., and W.D. maintained the data and conducted the statistical analysis. J.Y.W. and W.D. accessed and verified the raw data. The first draft of the manuscript was completed by Y.C.L., M.J., J.Y.W., and W.D. Y.L.C. and M.J. contributed equally. J.Y.W. and W.D. are the guarantors. All authors thoroughly reviewed and approved the final manuscript.

## CONFLICT OF INTEREST STATEMENT

The authors declare that they have no conflicts of interest.

## ETHICS STATEMENT

All procedures were followed in accordance with the ethical standards of the Helsinki Declaration. This examination was granted the approval of the Ethics Committee of Renji hospital (School of Medicine, Shanghai Jiao Tong University) (Approval Number: KY2019‐138 for human specimen, KY2021‐199‐B for animal). All patients offered their written informed consent.

## Supporting information

Supporting Information

## Data Availability

Data associated with the article are available from the corresponding author upon reasonable request.
