# Supplementary material for: Erucic acid improves the progress of pregnancy complicated with systemic lupus erythematosus by inhibiting the effector function of CD8+ T cells
Source: MedComm (2020). 2023 Sep 26;4(5):e382. doi: 10.1002/mco2.382 (PMC10522964; doi:10.1002/mco2.382)
Supplement: Supplementary file 1 — Supporting Information [file MCO2-4-e382-s001.docx]

**Erucic acid improves the progress of pregnancy complicated with systemic lupus erythematosus by inhibiting the effector function of CD8^+^ T cells**

Yanling Chang^1,2#^, Meng Jiang^1,2#^, You Wang^1,2^, Qiong Fu^4,5^, Sihan Lin^1,2^, Jiayue Wu^1,2^*^*^*, Wen Di ^1,2,3^*^*^*

^#^YL.C and M.J contributed equally

^1^Department of Obstetrics and Gynecology, Renji Hospital, School of Medicine, Shanghai Jiao Tong University, Shanghai 200127, China

^2^Shanghai Key Laboratory of Gynecologic Oncology, Shanghai 200127, China

^3^State Key Laboratory of Oncogenes and Related Genes, Shanghai Cancer Institute, Renji Hospital, School of Medicine, Shanghai Jiao Tong University

^4^Department of Rheumatology, Renji Hospital, School of Medicine, Shanghai Jiao Tong University, Shanghai 200127, China

^5^Shanghai Institute of Rheumatology, Shanghai 200001, China

^*^Correspondence: janet_wu_jiayue@163.com and Wen Di Email: diwen163@163.com

**Supplementary Table**

**Table S1**. Demographics tables of HPC and patients with pSLE

|  | HPC (*n* = 62) | pSLE (*n* = 90) | *p*-value |
| --- | --- | --- | --- |
| Maternal age (*years*) | 32.29 ± 3.67 | 30.58 ± 3.67 | 0.005^**^ |
| Gravidity (*N*) | 1.84 ± 0.94 | 2.01 ± 1.20 | 0.325 |
| Parity (*N*) | 1.31 ± 0.50 | 1.17 ± 0.40 | 0.070 |
| Anti-dsDNA (n; %) | NA | 83(92.2) | NA |
| Anti-Ro/SSA (n; %) | NA | 39(43.3) | NA |
| Anti-LA/SSB (n; %) | NA | 6(6.7) | NA |
| Anti-Sm (n; %) | NA | 21(23.3) | NA |
| aPL (n; %) | NA | 30(33.3) | NA |
| Medication |  |  |  |
| Glucocorticoid (n; %) | NA | 88(97.8) | NA |
| Hydroxychloroquine (n; %) | NA | 81(90.0) | NA |
| Immunosuppressive agent (n; %) | NA | 21(23.3) | NA |
| Aspirin (n; %) | NA | 46(51.1) | NA |
| LMWH (n; %) | NA | 10 (11.1) | NA |
| Antihypertensive agent (n; %) | NA | 11(12.2) | NA |
| Pregnancy outcome |  |  |  |
| Gestational weeks of delivery (*weeks*) | 39.23 ± 1.36 | 36.91 ± 2.31 | < 0.001^***^ |
| Preeclampsia (n; %) | 0 | 7(7.78) | 0.042^*^ |
| Preterm birth (n; %) | 2 (3.23) | 32 (35.56) | < 0.001^***^ |
| Fetal growth restriction (n; %) | 0 | 7 (7.78) | 0.042^*^ |
| Birth weight (*g*) | 3336.07 ± 403.29 | 2727.72 ± 611.38 | < 0.001^***^ |

Date are mean ± SD

NA= Not applicable

^*^*p*<0.05, ^**^*p*<0.01, ^***^*p*<0.001

**Table S2.** Dosage of glucocorticoid and hydroxychloroquine in patients with pSLE

|  | Glucocorticoid (mg) | Hydroxychloroquine (g) |
| --- | --- | --- |
| pSLE-1 | 10 | 0.2 |
| pSLE-2 | NA | 0.1 |
| pSLE-3 | 20 | 0.3 |
| pSLE-4 | 20 | 0.3 |
| pSLE-5 | 12.5 | 0.4 |
| pSLE-6 | 30 | 0.2 |
| pSLE-7 | 17.5 | 0.4 |
| pSLE-8 | 10 | 0.2 |
| pSLE-9 | 12.5 | 0.2 |
| pSLE-10 | 15 | 0.3 |
| pSLE-11 | 40 | 0.4 |
| pSLE-12 | 20 | 0.3 |
| pSLE-13 | 15 | 0.2 |
| pSLE-14 | 40 | 0.3 |
| pSLE-15 | 10 | 0.3 |
| pSLE-16 | 20 | 0.4 |
| pSLE-17 | NA | 0.2 |
| pSLE-18 | 5 | 0.2 |
| pSLE-19 | 15 | 0.4 |
| pSLE-20 | 12.5 | 0.2 |
| pSLE-21 | 10 | 0.2 |
| pSLE-22 | 10 | 0.1 |
| pSLE-23 | 10 | 0.1 |
| pSLE-24 | 2.5 | 0.2 |

**Supplementary Figure**

Figure S1


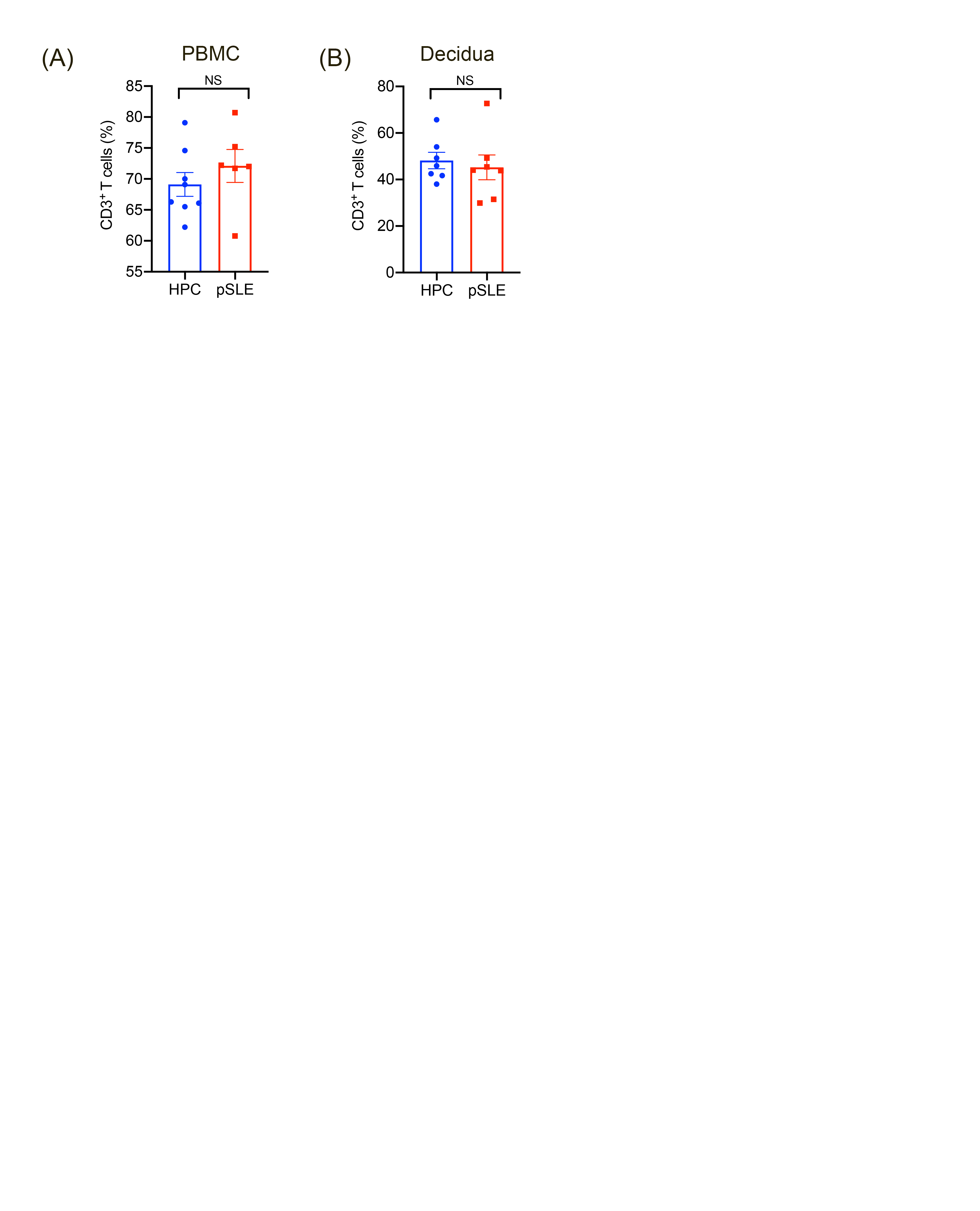


**Figure S1.** The frequencies of CD3^+^ T cells in HPC and patients with pSLE. (A) The frequencies of peripheral blood CD3^+^ T cells in HPC (*n* = 8) and patients with pSLE (*n* = 6). (C, D) The frequencies of decidua CD3^+^ T cells in HPC (*n* = 7) and patients with pSLE (*n* = 7). NS, not significant.

Figure S2


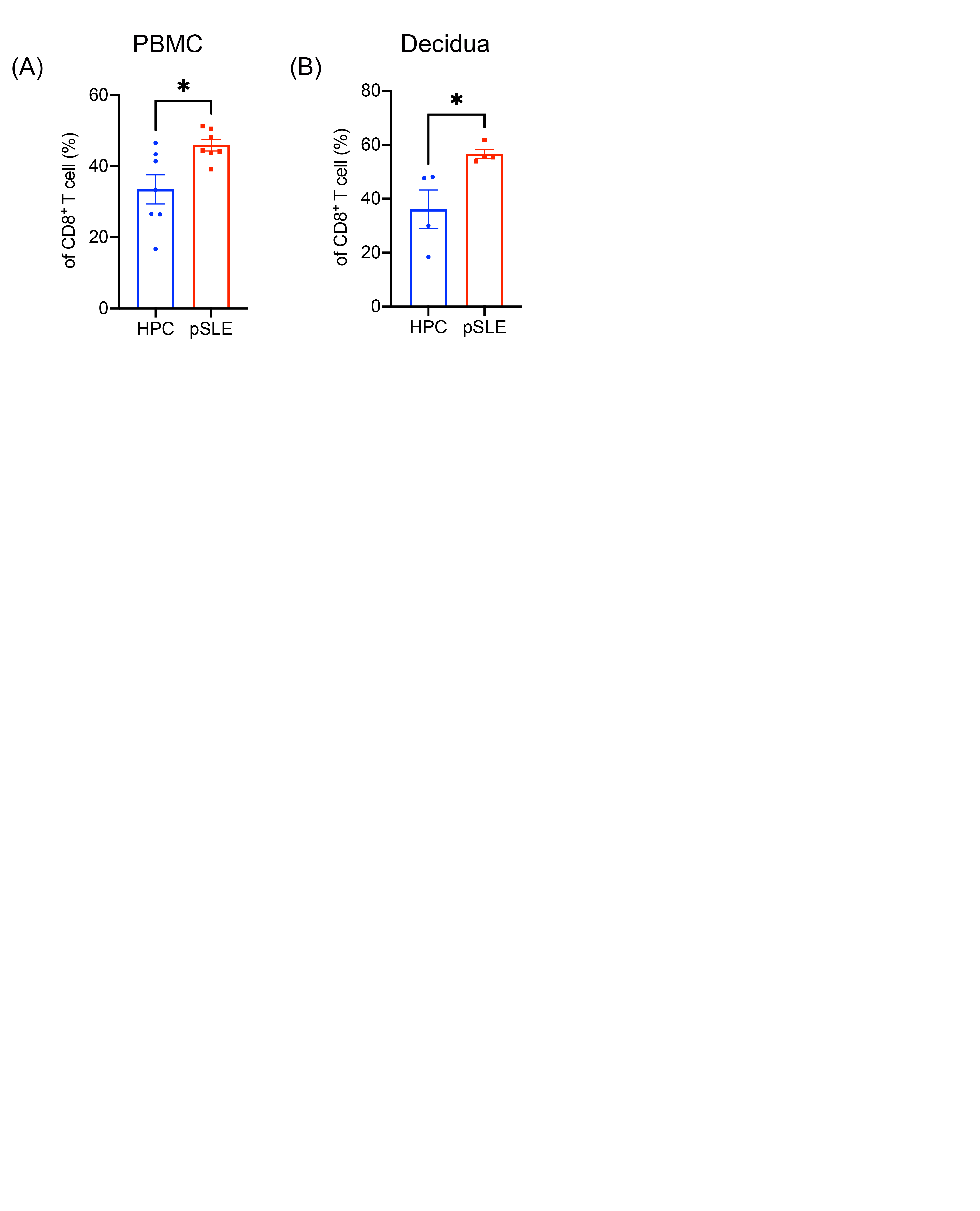


**Figure S2.** The proportion of CD8^+^ Teff cells in HPC and patients with pSLE. (A) The proportion of peripheral blood CD8^+^ Teff cells in HPC (*n* = 7) and patients with pSLE (*n* = 7). (B) The proportion of decidua CD8^+^ Teff cells in HPC (*n* = 4) and patients with pSLE (*n* = 4). ^*^*p* < 0.05.

Figure S3


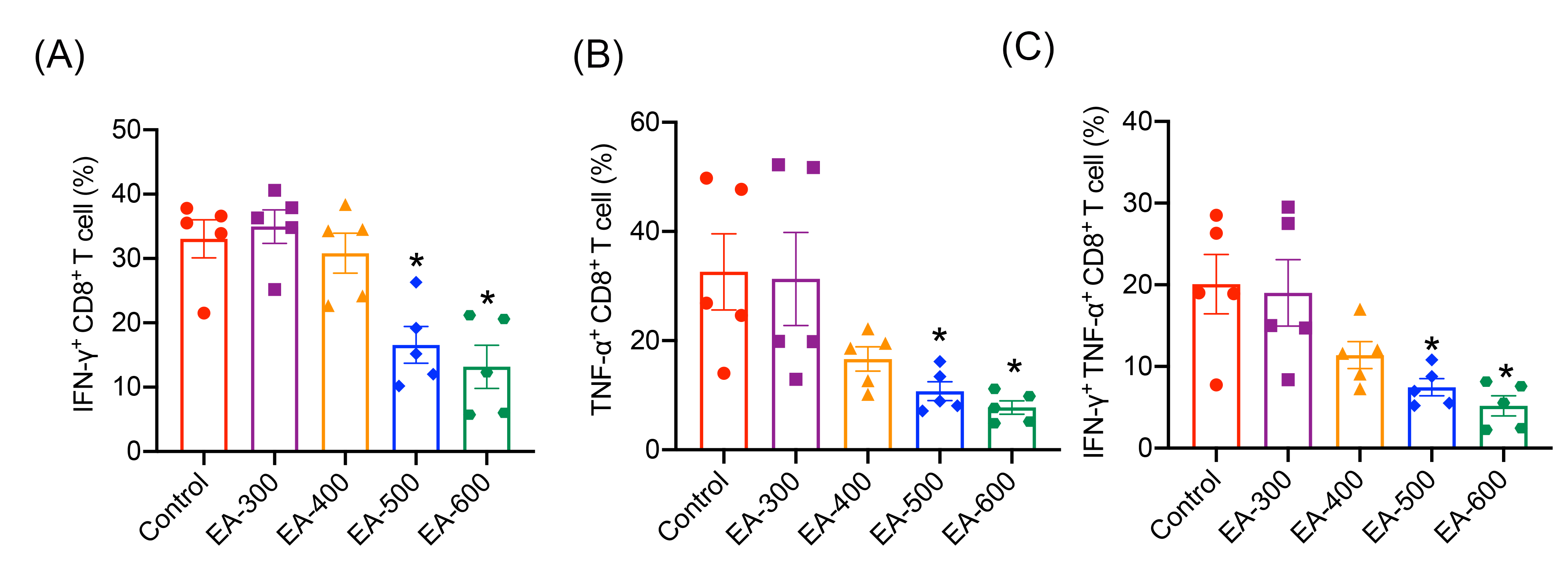


**Figure S3.** The inhibitory effect of different EA concentrations (0 as control, 300μM, 400μM, 500μM, 600μM) on inflammatory cytokines produced by CD8^+^ T cells at 24h. (A-C) IFN-γ, TNF-α and IFN-γ^+^ TNF-α^+^ CD8^+^ T cells produced by CD8^+^ T cells. ^*^*p* < 0.05.

Figure S4


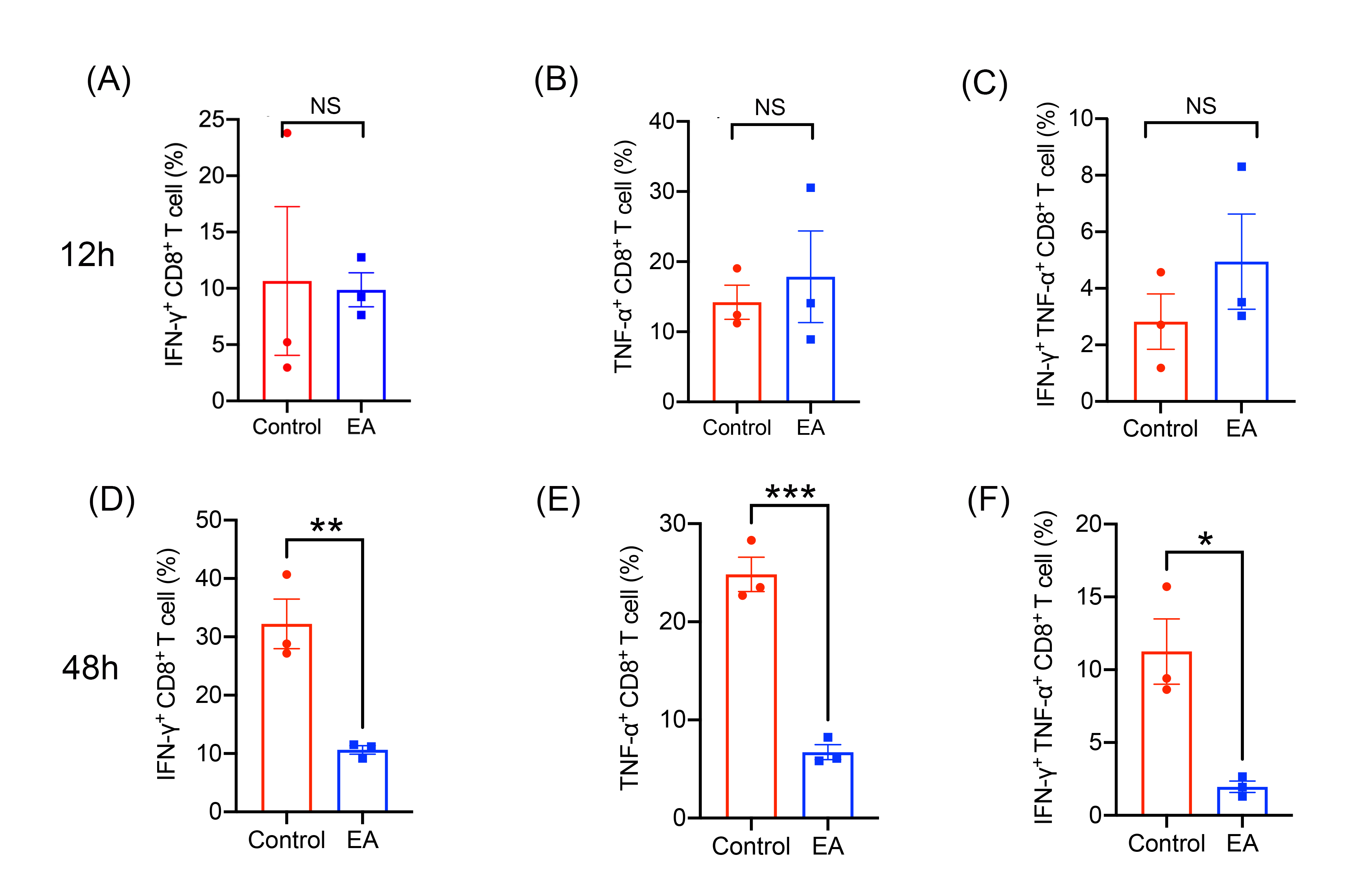


**Figure S4.** The inhibitory effect of EA on inflammatory cytokines produced by CD8^+^ T cells at different time. (A-C) IFN-γ, TNF-α and IFN-γ^+^ TNF-α^+^ CD8^+^ T cells produced by CD8^+^ T cells treated with EA for 12h. (D-F) IFN-γ, TNF-α and IFN-γ^+^ TNF-α^+^ CD8^+^ T cells produced by CD8^+^ T cells treated with EA for 48h. ^*^*p* < 0.05, ^**^*p* < 0.01, ^***^*p* < 0.001.

Figure S5


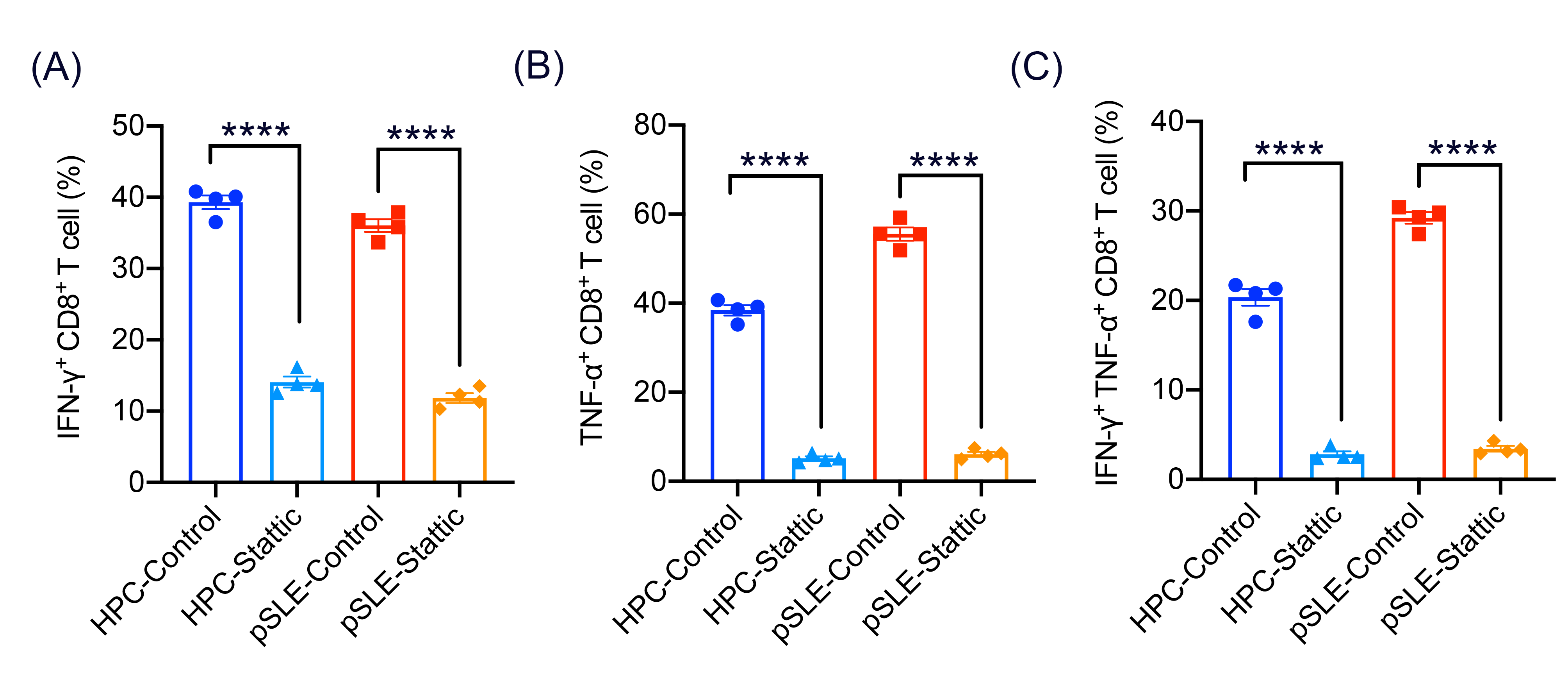


**Figure S5.** The inhibitory effect of Stattic (5μM) on inflammatory cytokines produced by CD8^+^ T cells at 24h. (A-C) IFN-γ, TNF-α and IFN-γ^+^ TNF-α^+^ CD8^+^ T cells produced by CD8^+^ T cells. ^****^*p* < 0.0001.

Figure S6


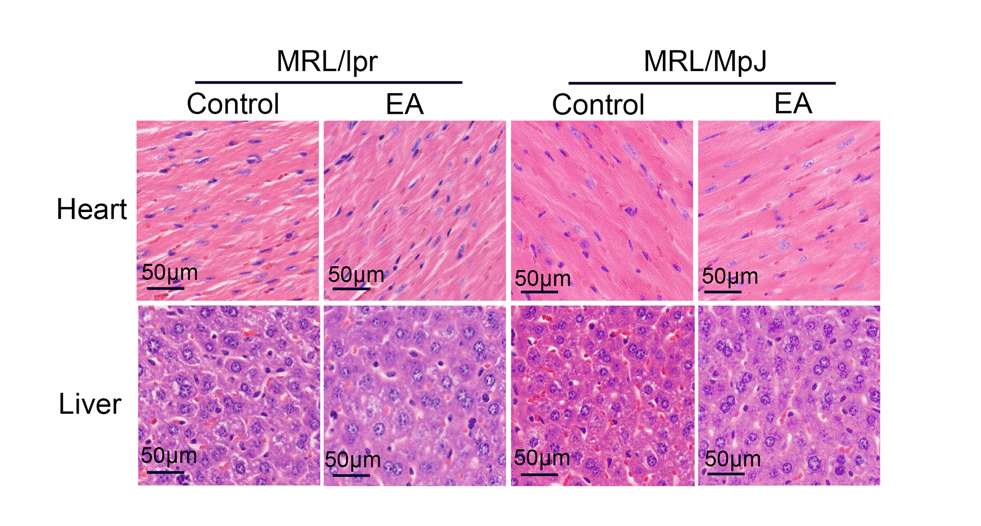


**Figure S6.** The effect of EA in pregnant MRL/lpr mice. H&E staining of heart and liver from pregnant mice. Scale bar = 50µm.
